# *Stevia rebaudiana* Bertoni, an American plant used as sweetener: Study of its effects on body mass control and glycemia reduction in Wistar male and female rats

**DOI:** 10.1371/journal.pone.0298251

**Published:** 2024-02-27

**Authors:** Samuel Mendoza-Pérez, Itzel Orta-Méndez-y-Sánchez, Rolando Salvador García-Gómez, Guillermo Ordaz-Nava, María Isabel Gracia-Mora, Lucía Macías-Rosales, Héctor A. Rico-Morales, Gerardo Salas-Garrido, María del Carmen Durán-Domínguez-de-Bazúa

**Affiliations:** 1 Faculty of Chemistry, Department of Chemical Engineering, *UNAM*, Laboratories of Enviromental Chemical Engineering and Chemistry, Mexico City, Mexico; 2 Department of Nutrition Physiology, Molecular Nutrition Area, National Institute of Medical Sciences and Nutrition “Salvador Zubirán”, *INCMNSZ*, Mexico City, Mexico; 3 Faculty of Chemistry, *UNAM*, Animal Experimentation Unit, *UNEXA*, Complex E, Circuito de la Investigación Científica s/n, Ciudad Universitaria, Mexico City, Mexico; 4 Faculty of Veterinary Medicine & Zootechny, Department of Pathology, *UNAM*, Circuito de la Investigación Científica s/n, Ciudad Universitaria, Mexico City, Mexico; Garraham Pediatric Hospital (Hospital de Pediatria Prof. Dr. Juan Garraham), ARGENTINA

## Abstract

*Stevia rebaudiana* Bertoni water extracts have been used as a natural sweetener and customary medicine by the indigenous inhabitants of South America for several hundred years. This plant was sent to Europe in the 16th century and was described by Peter Jacob Esteve in Spain. Recently the food industry has started to employ *S*. *rebaudiana* as sweetener using its glycosides after purification. Advertisement claims that Stevia glycosides is good for controling body mass and reducing glycemia. This study’s objective was to evaluate the effect of *S*. *rebaudiana* leaf extract on Wistar rats as animal model to prove its effectiveness on body mass control, glycemia reduction, and other biochemical parameters. Three groups were randomly formed with 24 males and 24 females: A blank group without any sweetener, a control group drinking water with 10% glucose, and the test group ingesting a 0.94% water extract of *S*. *rebaudiana*. Body mass measurements as well as food and drink consumption were daily performed. The experiment lasted 120 days after the specimens were weaned and got used to eating solid food. Euthanasia was done and blood serum was collected to evaluate the following biochemical parameters: Glucose, triglycerides, cholesterol, insulin, glucagon, leptin, ghrelin, and glucose-dependent insulinotropic peptide, GIP. Results indicated that only female rats had statistical differences in body mass gain. No relevant effects either positive or negative were found in the biochemical parameters measured. The crude extracts of *S*. *rebaudiana* did not show any relevant changes in biochemical and hormonal profiles, changes nor body mass with respect to the blank and control groups of young and healthy rats in the age range of infancy to youth. According to the results obtained, the therapeutic properties that have been associated to *S*. *rebaudiana* consumption especially for body mass control and glycemia reduction, did not occur in young and healthy male and female rats in equivalent age to infants, young children, and youths.

## Introduction

*S*. *rebaudiana* is native of northeastern Paraguay in South America. Its name in the guarani language is ka’a he’ẽ from ka’a (herb) and he’ẽ (sweet) [1]. Some samples were analyzed by Peter Jacob Esteve in the 16th century and recorded in his book *Hippocrates Coi Medicorum omnium principis epidemion liber secundus* a Petro Iacobo Steve *medico latinitate donatus et fusissimis commentariis illustratus*, *adjecta et singulis sententiis Graeca veritate*, *quo facilius diligens lector quanta sit servata fides intelligere possit*, written in Valencia in 1551 [2]. Mosè Giacomo Bertoni, a Swiss botanist, also described it in 1887, and in 1900 the Paraguayan chemist Ovidio Rebaudi isolated its sweet compounds. With the combination of the names of these three scientists, the scientific name of this plant was created. Starting with Stevia in honor of Petro Iacobo Stevi, then rebaudiana in honor of Ovidio Rebaudi and finally Bertoni in honor of Mosè Giacomo Bertoni [3].

*S*. *rebauiana* has been used by *Pãi Tavyterã* indigenous people in Paraguay for centuries. Its extract has been used as a natural sweetener and customary medicine by the indigenous inhabitants of South America for several hundred years [4,5]. The traditional method of use by the Paraguayan Guarani Indians was to dry the leaves and to use them to sweeten tea and medicines or to chew the leaves as a “sweet treat”. Stevia has also been used to enhance the taste of the Argentinian typical beverage “mate”, a bitter infusion [6].

Stevia has been proposed as a substitute of sugar from *Saccharum officinarum* [7]. It has acquired relevance at economic level although there is some controversy about its use [8]. Its cultivation has been extended through several countries such as Brazil, China, India, Japan, Korea, Mexico, Singapore, Taiwan, Thailand, among others [8,9].

Sweet taste is due to the high level of secondary metabolites such as diterpene glycosides present in leaves. Steviol is the aglycone of steviol glycosides. The remaining glycosides are its derivatives depending upon the substituents [8]. Although *S*. *rebaudiana* leaves contain more than 20 steviol glycosides, about 90% of its total content was stevioside and rebaudioside A, present in concentrations of 5.8±1.3 (w/w) and 1.8±1.2% (w/w) of the stevia leaves, respectively [10]. Sweetness depends upon the glycoside, for example stevioside is 100 to 270 times sweeter than sucrose whereas rebaudioside A is 150 to 320 times sweeter than sucrose [10].

Steviol glycosides are extracted from dry leaves of *S*. *rebaudiana* using either hot or cold water and concentrated using exchange resins [11]. Concentration of glycosides is done by exchange resins. These resins are washed with either methanol or ethanol and the resulting glycosides are recrystallized from the alcoholic solution. Purification of the glycosides is made with ionic exchange resins with a last step of spray drying. Some commercial products have around 99% of rebaudioside A. Although some companies use mixtures of steviosides, the products have at least 95% of any of the steviosides [12].

Steviol glycosides were approved by the Joint FAO/WHO Expert Committee on Food Additives [13] with an acceptable daily intake (ADI) of 4 mg steviol glycosides /kg body mass. The European Union accepted these compounds in December 2011 with an identical ADI value. The U. S. Food and Drug Administration (FDA) likewise established an ADI of 4mg/kg body mass for steviols with a purity of 95% [12,14–16].

Among the therapeutic uses attributed to *Stevia rebaudiana* are the antihyperglycemic potential [17,18] and the hipotensor effect attributed to its extracts and infusions [19,20]. Other authors have reported diuretic effects [3,21] and anticancerous effects to *Stevia rebaudiana* extracts [22] in rats induced with Ehrlich-Lettre ascites carcinoma (EAC), also known as Ehrlich cell. Antidiarrhea properties have also been reported [23]. However, the mechanisms of these effects are still not fully comprehended.

Furthermore, according to FDA, the use of stevia leaf and crude stevia extracts is not considered GRAS (in full) and their import into the United States is not permitted for use as sweeteners since these substances have not been approved as food additives [24]. Some literature reports indicate alterations in glucose levels in blood and effects in the reproductive, cardiovascular, and renal systems [3,17,20,25–30]. In 2019, the FDA [24] established that highly pure steviol glycosides derived from *S*. *rebaudiana* leaves, with a purity of at least 95% glycosides, have been recognized as safe for specific conditions of use. These safety assessments are documented in the Generally Recognized as Safe (GRAS) notice inventory. The Acceptable Daily Intake (ADI), determined by the Joint FAO/WHO Expert Committee on Food Additives (JECFA), is set at 4 milligrams per kilogram of body mass per day (mg/ kg body mass per day). However, it is noteworthy that the petitions submitted to the FDA did not provide sufficient data and necessary information to conclusively establish the safe use of stevia leaf, or its crude extract as ingredients in food products. This underscores the importance of comprehensive safety data in the regulatory assessment of food additives.

The research group in the 2020 study [11], the equisweetness between *S*. *rebaudiana* and sucrose was tested. Furthermore, in the 2021 study [31] that non-nutritive sweeteners, when consumed alongside a balanced diet, do not cause significant increases in body mass. However, in the 2022 research [32], the group found that saccharin and nutritive sweeteners caused alterations in some glycolytic and lipogenic enzymes. Given these possible positive properties of *S*. *rebaudiana* sweet compounds and the possible negative effects of the other components present in its dry leaves (secondary glycosides, flavonoids, phenolic compounds and essential oils), the objective of this study was to investigate the effects of daily consumption of *S*. *rebaudiana* infusions on various factors. These weres body mass gain and blood serum levels of glucose, triglycerides, cholesterol, insulin, glucagon, leptin, ghrelin, and glucose-dependent insulinotropic peptide (GIP). This research involved Wistar male and female rats over a 120-day period, starting after the rats had transitioned to solid food. The effects of consuming stevia infusions were compared against the effects of consuming drinks with glucose or without any sweetener.

## Materials and methods

### Water-stevia infusion preparation

**Fig 1:**
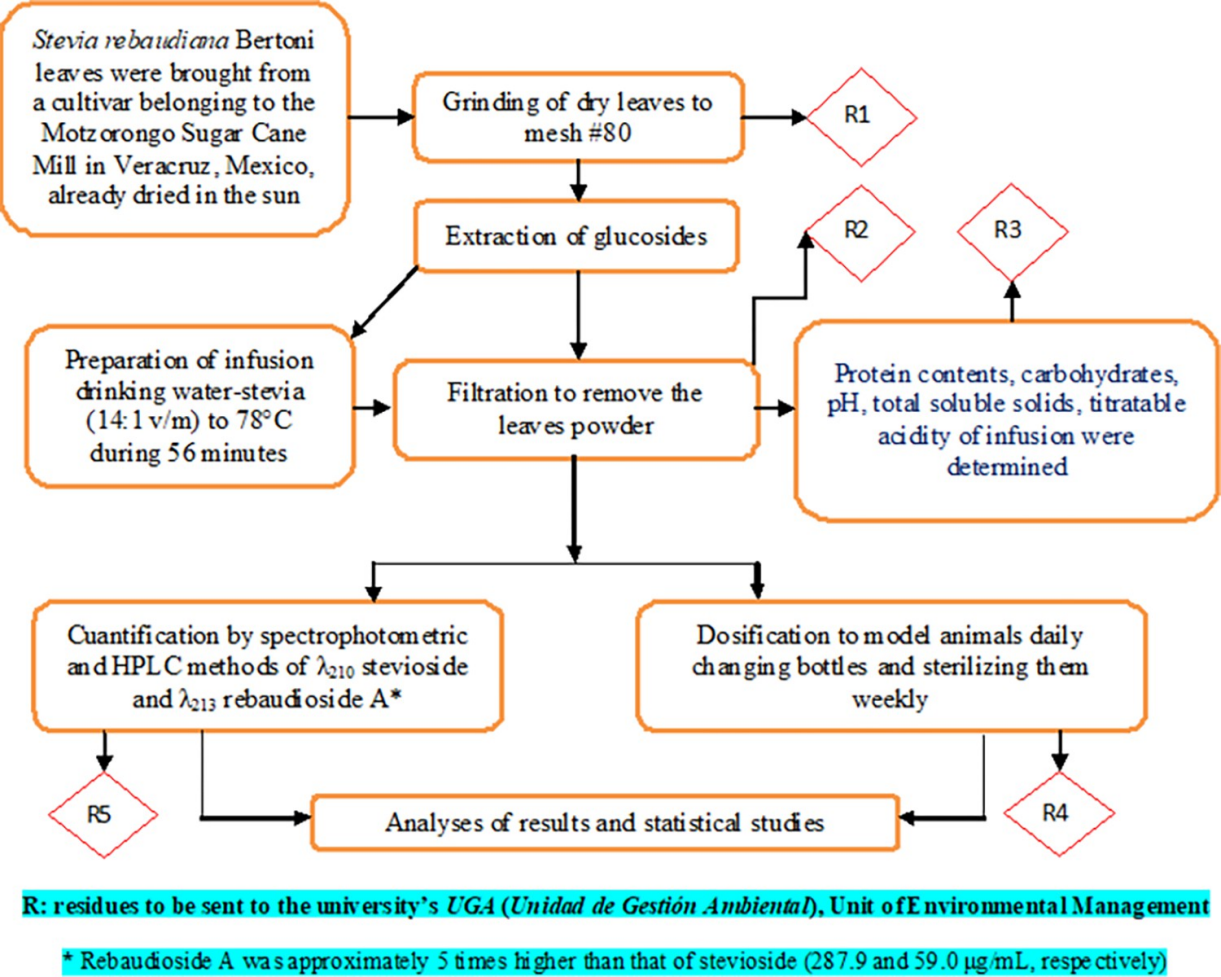
Methodology for preparing infusions and quantification of glucosides of interest (modified of [34]).

The standardized method of preparation of stevia infusion is briefly described: Dried leaves of *S*. *rebaudiana* (grown, harvested, and dried in the state of Veracruz, Mexico) were ground to a particle size of ≤177 μm. Daily, powder was added to boiling drinking water (92±2°C, for Mexico City atmospheric pressure boiling temperature), maintaining this temperature for 5 minutes (*S*. *rebaudiana* 0.94% m/v). The infusion was left to cool and settling of the powder reaching ambient temperature (20±5°C). Supernatant was measured to provide 250 mL to each clean glass bottle [33]. The diagram is shown in Fig 1 [34].

### Animal model

Wistar male (n = 24) and female (n = 24) rats weaned to consume solid food, were used for this study. Their body mass was between 35 to 45 g and the study was conducted for 120 days. *Ad libitum* solid diet was provided by Envigo™ company (Indianapolis, IN, United States) [35]. The food “*pellets”*, 100 g, were deposited in the external metal grid container. Leftovers were measured to calculate the amount of food ingested daily. If some spill was found it was also considered. They were randomly distributed in three groups (n = 8 per group and sex). One group drank an infusion of dry leaves of *S*. *rebaudiana* (STE). The second group drank water sweetened with glucose (GLU) as control. A third blank group (BLK) drank plain water. The specimens were individually located in sterilizable polysulfonate cages, in a room with controlled relative humidity (between 65–70%), ventilation, and temperature (22±1°C) maintaining 12 hours light and 12 hours dark conditions. Cages dimensions were 42.5 x 26.6 x 20 cm during the 120 days of experimentation. The rats daily had a period of game outside of the cages during weighing, when the cages were cleaned, and the food and drink measured and the containers changed by clean ones. Each specimen was marked in the ear for identification. The principles established in the Guide for the Care and Use of Laboratory Animals [36], that are similar to the Mexican legislation [37], were followed. The protocol was authorized by the Institutional Committee for the Care and Use of Laboratory Animals (*Comité Institucional para el Cuidado y Uso de los Animales de Laboratorio*, *CICUAL*, in Spanish) of the Faculty or College of Chemistry (*Facultad de Química*, *FQ*, in Spanish) of the National Autonomous University of Mexico (*Universidad Nacional Autónoma de México*, *UNAM*, in Spanish), Mexico. Drinks daily given had the following concentrations: Dry leaves of *Stevia rebaudiana*, 0.94%, prepared according to a standardized method [34]; glucose (Sigma-Aldrich, Mexico City, Mexico) used as control, 10%; and plain drinking water without any sweetener as blank. These sweeteners concentrations were determined as the equivalent of a sucrose solution of 10% taken as a reference (for the soft drinks that contain sucrose by a panel of 20 untrained judges, between 20 and 23 years old) [11]. Drinks were given without any restriction of time or quantity (*ad libitum*). Every 24 hours the amount consumed was quantified. The glass bottles and “straws” were changed by clean ones with new drink. The washed bottles and straws were sterilized to avoid any contamination once a week. As already mentioned, in a daily basis, the model animals were weighed at the same time of the day and in the same order in an electronic precision balance (SPE601, OHAUS^™^ de Mexico SA de CV, Mexico City, Mexico). Cages were carefully monitored for any evidence of spillage, and crumbs were considered for the control of food intake. The water intake was also determined subtracting the remaining amount of the daily supplied amount (250 mL). During this time of the day the rats were left to play together. Theoretically calculated energy intake was obtained adding energy equivalent per gram of feed daily ingested per rat. For the two groups that consumed the stevia infusion, the energy equivalent was considered negligible, taking only the amount per gram of feed. The same occurred for the blank groups, only solid food consumption was considered. The energy contribution of Teklad Global 2018S diet was 13 kJ/g [35]. For the two groups that ingested glucose the calculation was carried out adding the energy provided by feed as well as by the drinks. The energy contribution of glucose was 1.67 kJ/mL [38]. The cumulative food intake, water intake, and calculated energy intake taken daily were uploaded to an excel sheet. Cumulative body mass gain was calculated by the subtraction of the basal body mass from the mass obtained every day.

### Euthanasia

At the end of 120 days period was over, after a 12 h fasting time, euthanasia was carried out. Each rat was placed individually in a chamber with air rich in CO_2_ (70% minimum). Once asleep they were decapitated with a guillotine for rodents. All experimental procedures were conducted in accordance with guidelines of the National Research Council for the Care and Use of Laboratory Animals [36], and the respective Mexican legislation: Official Mexican Standard [37]. Internal organs were collected for future research. Blood samples were also collected in specific tubes with a separator gel (SST 368159, BD Vacutainer, Becton and Dickinson, Burlington, NC, United States) with the aid of individual polypropylene funnels. The tubes were left to stand for 30 minutes to allow clotting, and then the tubes were centrifuged at 2100 *g* force (3000 rpm) for 15 minutes at 25°C. The serum collected was stored in 2 mL Eppendorf tubes (Hamburg, Germany), and immediately frozen at -70°C for later analysis. Insulin, glucagon, leptin, ghrelin, and glucose dependent insulinotropic peptide, GIP, were calculated by interpolation on the corresponding standard curves simultaneously determined by fluoroimmunoassays quantifying the mean fluorescence emitted by Luminex XMAP technology (MAGPIX®, LUMINEX). These fluoroimmunoassays are commercially available as kits provided by Millipore™ (Merck-Millipore Corporation, Billerica, MA, United States). For this purpose, it was used the Milliplex MAP Kit Rat Metabolic Magnetic Bead Panel Cat. # RMHMAG-84K. Serum glucose, triglyceride, and total cholesterol levels were determined using the automatic analyzer Cobas C111 of Roche^™^ brand (Roche, Rotkreuz, Switzerland). All analyses were performed in triplicate.

### Statistical analysis

Statistical analysis was done using the software StatGraphics Centurion XVI (StatPoint Technologie, Inc, Warrengton, VA, United States), and graphs were produced by using the software GrandPad Prism 6 (GraphPad Software Inc., La Jolla, CA, United States). The experimental design was multifactorial involving two categorical factors: a) sweetener factor and b) sex factor. The experimental data were evaluated by analysis of variance (ANOVA) of one way or two ways. The Duncan test was used as a "*post hoc*" test to identify the differences between groups. The normality and homoscedasticity of data sets were verified with the Shapiro-Wilk test and the Levene test, respectively. In those cases, in which there was no homoscedasticity the Welch test was performed and as a multiple comparison the Games-Howell multiple range test was carried out. Reported values are means and confidence intervals (CI). The p<0.05 was taken as significant for all analyses.

## Results and discussion

### Cumulative body mass gain

**Fig 2:**
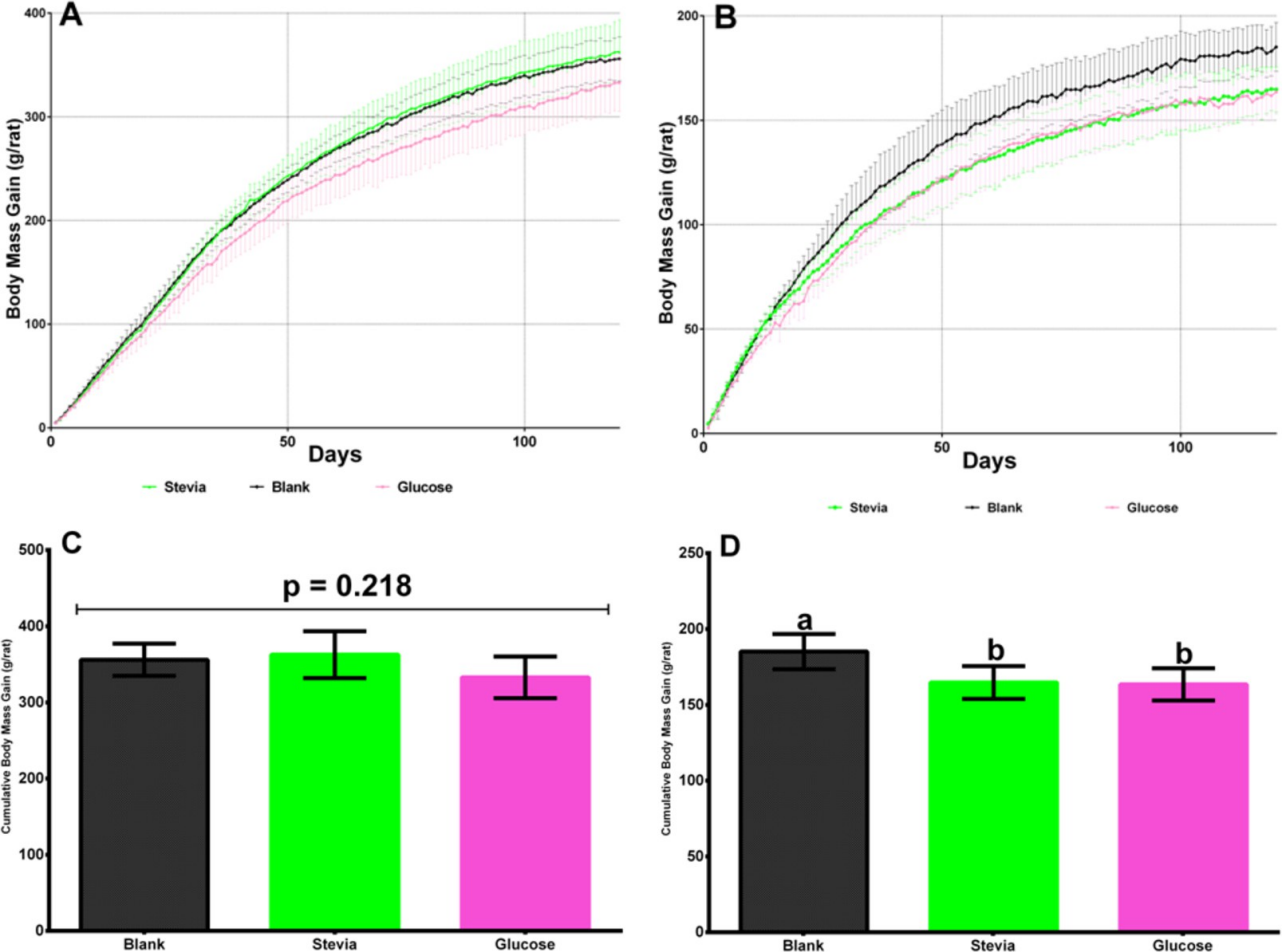
A. Male Wistar rats final cumulative body mass gain 120 days after weaning consuming a water infusion of *S*. *rebaudiana* dried leaves, drinking water with glucose (control), and plain water (blank). Each line represents the trend in the evaluated period where each point comprises the average body mass gain of the group and its CI at 95%, n = 8. B. Female Wistar rats final cumulative body mass gain 120 days after weaning consuming a water infusion of *S*. *rebaudiana* dried leaves, drinking water with glucose (control), and plain water (blank). Each line represents the trend in the evaluated period where each point comprises the average body mass gain of the group and its CI at 95%, n = 8. C. Male Wistar rats statistical analyses of final cumulative body mass gain data 120 days after weaning consuming a water infusion of *S*. *rebaudiana* dried leaves, drinking water with glucose (control), and plain water (blank). The following letters indicate significant differences p<0.05: a, b, c, d. Duncan test. D. Female Wistar rats statistical analyses of final cumulative body mass gain data 120 days after weaning consuming a water infusion of *S*. *rebaudiana* dried leaves, drinking water with glucose (control), and plain water (blank). The following letters indicate significant differences p<0.05: a, b, c, d. Duncan test.

Among male Wistar rats no significant differences (p = 0.218) in cumulative data of body mass gain were found after 120 days of experimentation (Fig 2A and 2C).

For female rats there were significant intergroup differences (p = 0.0058) for cumulative body mass gain, both for those that drank the stevia infusion (158–171 g) and those that drank water sweetened with glucose (157–170 g) compared with the blank group (178–192 g) (Fig 2B and 2D). Naturally, there were significant differences between male and female rats (p<0.0001), presenting the male rats a higher body mass gain (339–358 *versus* 161–181 g for the female rats. This is a normal pattern for Wistar rats [39].

### Consumed food

**Fig 3:**
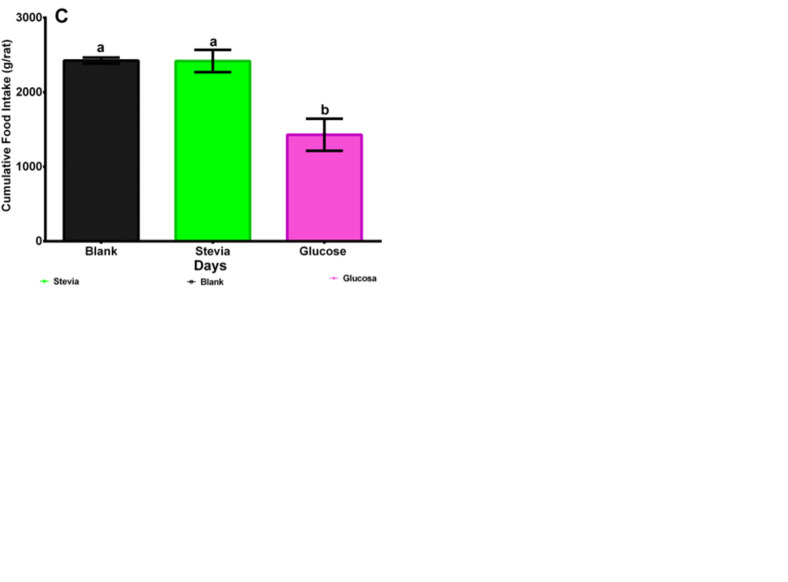
A. Male Wistar rats daily food intake during 120 days after weaning consuming stevia infusion, drinking water sweetened with glucose (control), and plain water (blank). Each line represents the trend in the evaluated period where each point comprises the average food intake of each group and its CI at 95%, n = 8. B. Female Wistar rats daily food intake during 120 days after weaning consuming stevia infusion, drinking water sweetened with glucose (control), and plain water (blank). Each line represents the trend in the evaluated period where each point comprises the average food intake of each group and its CI at 95%, n = 8. C. Male Wistar rats statistical analyses of final cumulative food intake data 120 days after weaning consuming stevia infusion, drinking water sweetened with glucose (control), and plain water (blank). The following letters indicate significant differences p<0.05: a, b, c, d. Duncan test. D. Female Wistar rats statistical analyses of final cumulative food intake data 120 days after weaning consuming stevia infusion, drinking water sweetened with glucose (control), and plain water (blank). The following letters indicate significant differences p<0.05: a, b, c, d. Duncan test.

Fig 3A shows average food daily consumed for male Wistar rats. Fig 3B data are for female Wistar rats. The same tendency can be observed in both cases: The two groups drinking water sweetened with glucose ate less food than the groups drinking plain water (blank) and the stevia infusion. In both cases (Fig 3C and 3D) there were significant differences, p<0.0001, concerning the total amount of consumed food. Additionally, significant differences, p<0.0001, existed between male and female rats’ cumulative food consumption, 2000–2190 g/male rat y 1330–1560 g/female rat, respectively.

### Drink ingested

**Fig 4:**
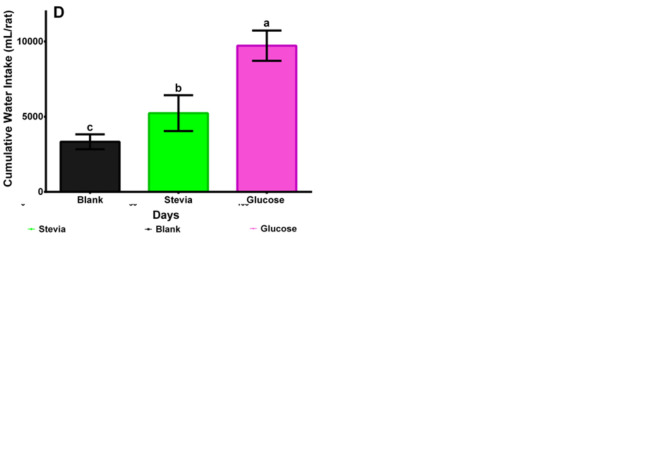
A. Male Wistar rats daily water intake during 120 days after weaning consuming stevia infusion, drinking water sweetened with glucose (control), and plain water (blank). Each line represents the trend in the evaluated period where each point comprises the average water intake of each group and its CI at 95%, n = 8. B. Female Wistar rats daily water intake during 120 days after weaning consuming stevia infusion, drinking water sweetened with glucose (control), and plain water (blank). Each line represents the trend in the evaluated period where each point comprises the average water intake of each group and its CI at 95%, n = 8. C. Male Wistar rats statistical analyses of final cumulative water intake data 120 days after weaning consuming stevia infusion, drinking water sweetened with glucose (control), and plain water (blank). The following letters indicate significant differences p<0.05: a, b, c, d. Duncan test. D. Female Wistar rats statistical analyses of final cumulative water intake data 120 days after weaning consuming stevia infusion, drinking water sweetened with glucose (control), and plain water (blank). The following letters indicate significant differences p<0.05: a, b, c, d. Duncan test.

Male and female Wistar rats (Fig 4A and 4B) drank significantly higher amount of water sweetened with glucose with respect to the blank and stevia infusion groups (Fig 4C). For female Wistar rats (Fig 4D) the three groups were significantly different (p<0.0001). Sex was a significant factor in the amount of drink ingested. In the female rats (Fig 4D), the rats drank significantly more stevia infusion than drinking water. In contrast, in male rats (Fig 4C) there was no significant difference in the amount of drink ingested between the drinking water and stevia infusion groups. The above indicates that female rats have a greater predilection for stevia infusions than males.

### Theoretically calculated ingested energy

**Fig 5:**
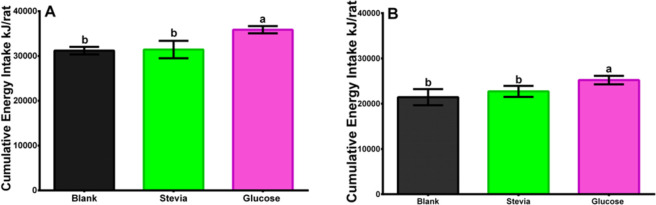
A. Male Wistar rats statistical analyses of theoretically calculated energy cumulative intake data 120 days after weaning consuming stevia infusion, drinking water sweetened with glucose (control), and plain water (blank). Its CI is at 95%. The following letters indicate significant differences p<0.05: a, b, c, d. Duncan test. B. Female Wistar rats statistical analyses of theoretically calculated energy cumulative intake data 120 days after weaning consuming stevia infusion, drinking water sweetened with glucose (control), and plain water (blank). Its CI is at 95%. The following letters indicate significant differences p<0.05: a, b, c, d. Duncan test.

Male Wistar rats (Fig 5A and 5B) theoretically ingested higher amounts of calories, particularly the group that consumed water sweetened with glucose with respect to their blank and stevia infusion groups. Also, due to the higher ingestion of food and their body mass increase, the male groups theoretically ingested more cumulative energy than their females counterparts, p<0.0001: 31,900–33,800 kJ/male rat and 22,700–24,400 kJ/female rat, respectively.

### Biochemical serum measurements

Blood serum concentrations of glucose, triglycerides, cholesterol, and the hormonal parameters (insulin, glucagon, leptin, ghrelin, and glucose dependent insulinotropic peptide, GIP) were determined at the end of the 120 days of experimentation after weaning and with a 12 h fasting.

### Glucose, triglycerides, and cholesterol

**Fig 6:**
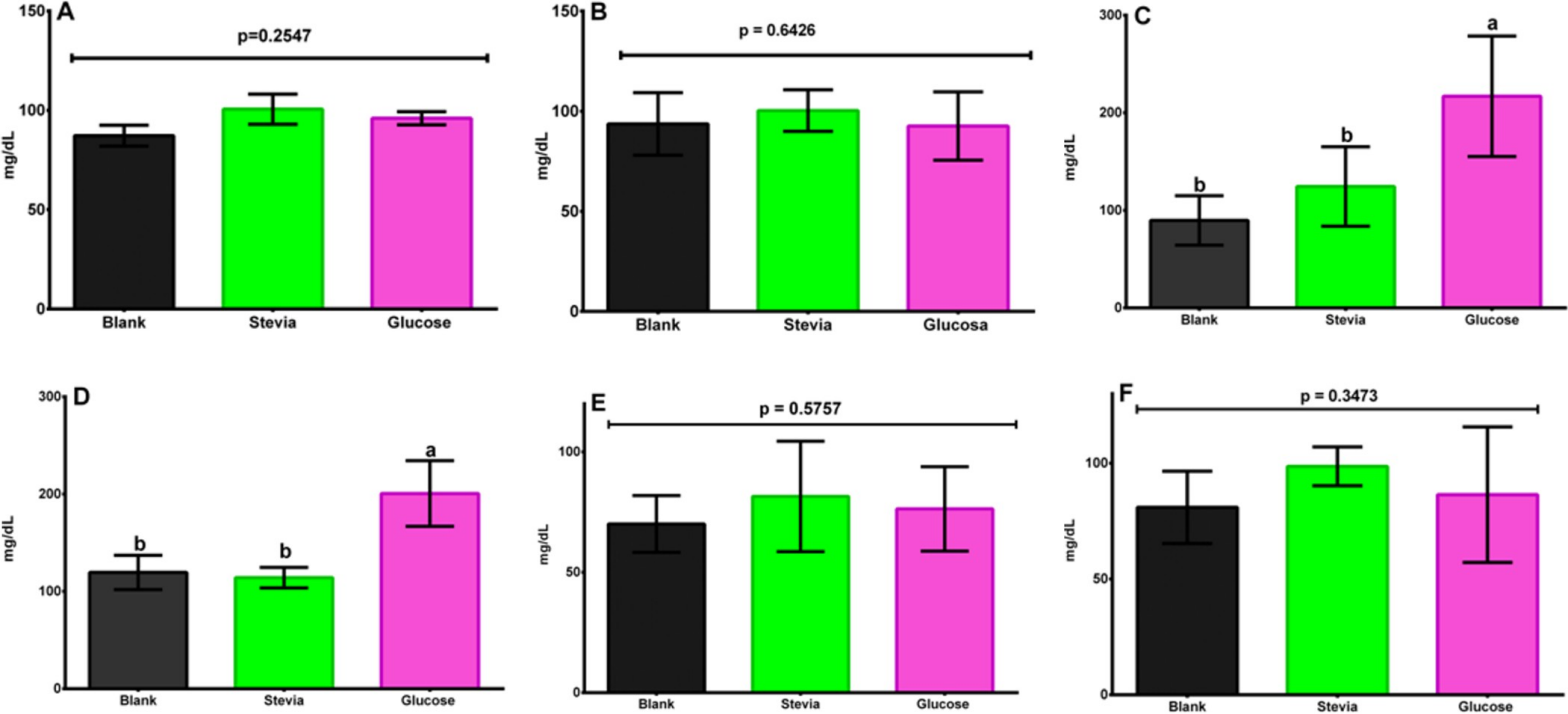
A. Male Wistar rats statistical analyses of blood serum glucose data 120 days after weaning consuming stevia infusion, drinking water sweetened with glucose (control), and plain water (blank). Its CI is at 95%. The following letters indicate significant differences p<0.05: a, b, c, d. Games-Howell Method n = 8. B. Female Wistar rats statistical analyses of blood serum glucose data 120 days after weaning consuming stevia infusion, drinking water sweetened with glucose (control), and plain water (blank). Its CI is at 95%. The following letters indicate significant differences p<0.05: a, b, c, d. Games-Howell Method n = 8. C. Male Wistar rats statistical analyses of serum triglycerides data 120 days after weaning consuming stevia infusion, drinking water sweetened with glucose (control), and plain water (blank). Its CI is at 95%. The following letters indicate significant differences p<0.05: a, b, c, d. Games-Howell Method n = 8. D. Female Wistar rats statistical analyses of serum triglycerides data 120 days after weaning consuming stevia infusion, drinking water sweetened with glucose (control), and plain water (blank). Its CI is at 95%. The following letters indicate significant differences p<0.05: a, b, c, d. Games-Howell Method n = 8. E. Male Wistar rats statistical analyses of serum cholesterol data 120 days after weaning consuming stevia infusion, drinking water sweetened with glucose (control), and plain water (blank). Its CI is at 95%. The following letters indicate significant differences p<0.05: a, b, c, d. Games-Howell Method n = 8. F. Female Wistar rats statistical analyses of serum cholesterol data 120 days after weaning consuming stevia infusion, drinking water sweetened with glucose (control), and plain water (blank). Its CI is at 95%. The following letters indicate significant differences p<0.05: a, b, c, d. Games-Howell Method n = 8.

No significant differences were found, neither among the three groups of the same sex nor among those of different sex (Fig 6A and 6B) for glucose in blood serum, p = 0.2547 and p = 0.6426, respectively. Additionally, no differences were found between male and female specimens (p = 0.8474).

For triglycerides results were different. There were intergroup differences for both male and female rats, p = 0.0003 (Fig 6C, male rats) and p = 0.0284 (Fig 6D female rats). The groups for male and female rats that drank water sweetened with glucose had the highest levels of triglycerides. These levels were above the acceptable values for this strain: 11.15 to 215.04 mg/dL [40], were almost the double as the levels for the stevia and blank groups [40].

No existing differences in blood serum levels of cholesterol among male and female rats were determined (p = 0.0591). Also, there were no intergroup differences in male Wistar rats (Fig 6E) and female rats (Fig 6F) with respect to this biochemical parameter.

### Insulin, glucagon, leptin, ghrelin, and glucose dependent insulinotropic peptide, GIP

**Fig 7:**
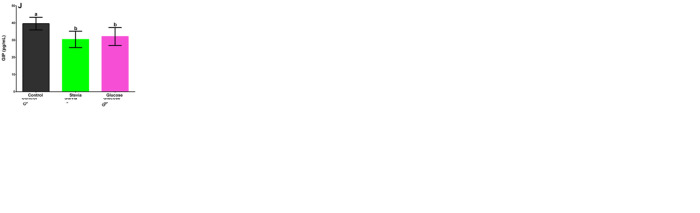
A. Male Wistar rats statistical analyses of serum insulin data 120 days after weaning consuming stevia infusion, drinking water sweetened with glucose (control), and plain water (blank). Its CI is at 95%. The following letters indicate significant differences p<0.05: a, b, c, d. Games-Howell Method n = 8. B. Female Wistar rats statistical analyses of serum insulin data 120 days after weaning consuming stevia infusion, drinking water sweetened with glucose (control), and plain water (blank). Its CI is at 95%. The following letters indicate significant differences p<0.05: a, b, c, d. Games-Howell Method n = 8. C. Male Wistar rats statistical analyses of serum glucagon data 120 days after weaning consuming stevia infusion, drinking water sweetened with glucose (control), and plain water (blank). Its CI is at 95%. The following letters indicate significant differences p<0.05: a, b, c, d. Games-Howell Method n = 8. D. Female Wistar rats statistical analyses of serum glucagon data 120 days after weaning consuming stevia infusion, drinking water sweetened with glucose (control), and plain water (blank). Its CI is at 95%. The following letters indicate significant differences p<0.05: a, b, c, d. Games-Howell Method n = 8. E. Male Wistar rats statistical analyses of serum leptin data 120 days after weaning consuming stevia infusion, drinking water sweetened with glucose (control), and plain water (blank). Its CI is at 95%. The following letters indicate significant differences p<0.05: a, b, c, d. Games-Howell Method n = 8. F. Female Wistar rats statistical analyses of serum leptin data 120 days after weaning consuming stevia infusion, drinking water sweetened with glucose (control), and plain water (blank). Its CI is at 95%. The following letters indicate significant differences p<0.05: a, b, c, d. Games-Howell Method n = 8. G. Male Wistar rats statistical analyses of serum ghrelin data 120 days after weaning consuming stevia infusion, drinking water sweetened with glucose (control), and plain water (blank). Its CI is at 95%. The following letters indicate significant differences p<0.05: a, b, c, d. Games-Howell Method n = 8. H. Female Wistar rats statistical analyses of serum ghrelin data 120 days after weaning consuming stevia infusion, drinking water sweetened with glucose (control), and plain water (blank). Its CI is at 95%. The following letters indicate significant differences p<0.05: a, b, c, d. Games-Howell Method n = 8. I. Male Wistar rats statistical analyses of serum glucose dependent insulinotropic peptide, GIP, data 120 days after weaning consuming stevia infusion, drinking water sweetened with glucose (control), and plain water (blank). Its CI is at 95%. The following letters indicate significant differences p<0.05: a, b, c, d. Games-Howell Method n = 8. J. Female Wistar rats statistical analyses of serum glucose dependent insulinotropic peptide, GIP, data 120 days after weaning consuming stevia infusion, drinking water sweetened with glucose (control), and plain water (blank). Its CI is at 95%. The following letters indicate significant differences p<0.05: a, b, c, d. Games-Howell Method n = 8.

Male Wistar rats (Fig 7A) had no intergroup difference for insulin levels (p = 0.406), and their levels were significantly higher than that of female Wistar rats, p<0.0001 (896–1070 pg/mL for male rats *versus* 651–823 pg/mL for female rats). The female rats (Fig 7B) showed differences in their insulin concentrations (p = 0.0349) among the three groups. Glucose control and stevia groups shared a non significant difference, whereas the stevia and blank groups also did not show significant difference in the insulin contents in blood serum. These were significantly higher than for female Wistar rats, p<0.0001, 896–1070 pg/mL for male rats *versus* 651–823 pg/mL for female rats.

The female specimens (Fig 7B) had differences in their insulin concentrations (p = 0.0349) among the three groups. Glucose control and stevia groups shared a non significant difference, whereas the stevia group specimens also shared with the blank group the insulin contents in blood serum. These results pose interesting further research questions as it will be further discussed with fairly recent literature reviews [9].

Wolever and Miller [41] established that the intake of sucrose or glucose creates a postprandial increase in blood glucose and insulin. Hence, the results obtained in this 120-day experiment are interesting since steviol glycosides influenced postprandial blood glucose and insulin concentrations [9].

For glucagon, no statistical differences were found neither between intergroups nor different sex specimens (Fig 7C and 7D). Not many references address this parameter but, according to the research carried out by Envigo researchers [40] these are normal values for the specimens in study, and these data are in concordance with the following ones: Light period (80.1±3.5 pg/mL) and the dark period (87.3±3.4 pg/mL), F = 14.91, P = 0.008 [42].

Circulating leptin concentrations for stevia animals group were higher in male Wistar rats than in their female counterparts (Fig 7E and 7F have different scale in the ordinates), p<0.0001. Besides, the interaction sex*sweetener was significant, p = 0.0359. These results are clearly appreciated since female rats had significant differences among them, p = 0.0087, glucose group with respect to the other two.

With respect to the hormone ghrelin, just like glucagon, no statistical differences were found neither between intergroups nor sex specimens (Fig 7G and 7H).

The glucose dependent insulinotropic peptide, GIP, had a similar behavior as insulin. Male Wistar rats (Fig 7I) had no significant differences among the three groups (p = 0.117). Female Wistar rats (Fig 7J) did have intergroup significant differences, p<0.0001. GIP blood serum levels were not significantly different between female rats (31.6–36.5 pg/mL) and male rats (30.1–35.1 pg/mL).

## Final discussion

Results in this research indicated that male Wistar rats that ingested *Stevia rebaudiana* dry leaves infusion with a concentration of 0.94% together with the diet had no significant differences with the other groups in body mass (326–401 g/male rat). Neither a positive effect in the reduction of body mass nor a negative one on the increase of body mass can be attributed to the consumption of daily infusion of *Stevia rebaudiana* dry leaves. In past literature reviews, the age of animal models was not stated, while the experimental period with humans is short that, the only thing that it can be said is that when “babies-children-youth” rats of both sexes are concerned, about 150 days old considering the weeks before weaning and the adaptation period to solid food [43], *S*. *rebaudiana* dry leaves infusions did not alter biochemical parameters in males. For female rats, the insulin, and the glucose dependent insulinotropic peptide, GIP, have significantly different values when compared with the control and blank groups [9].

Among the studied groups there were statistically differences in the amount of food ingested from the blank as it was shown in Fig 3A to 3D with glucose groups with the lowest ingestion. Boakes et al. [44], de-Matos-Feijó et al. [45], Foletto et al. [46], and Polyák et al. [47] reported that hypocaloric sweeteners did not alter the amount of food ingestion with respect to blank groups drinking plain water. These results do not agree with those observed by Anton et al. [48], with a very small group of humans during a three-day experiment. They concluded that the consumption of a snack sweetened with *Stevia rebaudiana*, produced a lower food intake for lunch and dinner meals. According to these reviews, the food or drink matrix seem to be relevant for studying the effects of *S*. *rebaudiana*. In future research this comparison should be assessed.

Contrary to what was observed on consumed food, the amount of water intake was statistically higher for glucose sweetened drinking water (Fig 4A and 4B) due to the inherent cravings for easy to digest energy sources in mammals. For male Wistar rats, there were no differences concerning the amount of water intake in the *Stevia rebaudiana* group with respect to the blank group. For the female Wistar rats the three groups were significantly different with *Stevia* group showing higher intake than the blank group. According to Samuel et al. [9] the absorption, metabolism, and excretion of steviol glycosides have been extensively reviewed by multiple scientific authorities and experts, including the European Food Safety Authority [49], and recently by Magnuson et al. [50]. Steviol glycosides are undigested in the upper gastrointestinal tract. They are hydrolyzed or degraded only when they come into contact with microbiota in the colon that cleave the glycosidic linkages, removing the sugar moieties, leaving behind the steviol backbone that is absorbed systemically, glucuronidated in the liver, and excreted via urine in humans and via feces in rats [50]. This indicates that gut microbial communities may be using glucids present in the molecules of the steviol glycosides just as the glucose in control group.

With respect to energy received by the studied groups through food and drinks, especially in the case of glucose control group, it is appreciated that the model animals adjust its consumption accordingly. Instinct plays a very important role concerning energy input in the organism. Two groups of researchers agree on this [51,52]. According to Swithers and Davidson [53] and Swithers [54], animals may use the sweet flavor of foods to predict the energy contents of foods. Interestingly, Swithers et al. [55] complement this idea indicating that sweet taste without any associated energy produces significant effects compared with sweet taste associated with energy, and as time goes by these effects may contribute to a positive energy balance and an increase in body mass. These concepts were supported by the research carried out by Wang et al. [56], suggesting a possible physiological response to this phenomenon. Wang et al. [56] indicated that a chronic consumption of an unbalanced sweet/calories diet, increase the neural responses to food craving of neuropeptide Y. This might be the reason why the sweet taste tongue buds associate its stimulation to an energy input, becoming then a need to readjust the energy input to compensate this taste signals to the brain. Maybe as this compensation is not carried out properly, a higher energy input occurs. Davidson et al. [57] concluded that caloric sweeteners consumption reduces the effectivity of the learning associated between sweet taste and energy liberation after food and/or drink ingestion. In this research a normal diet that had no sweet taste was employed and the results obtained seem to be supported by Davidson et al. [57] hypothesis. Predictive interferences between energy and sweet taste caused hypo-caloric sweeteners seem to be relevant only when a sweet diet is consumed after ingesting sweeteners, and thus, more research is needed to corroborate this apparent dependence of diet type and sweeteners effects.

About blood serum glucose, Suanarunsawat et al. [18] reported that neither crude extracts of *Stevia rebaudiana* or the stevioside had an impact in seric levels of glucose for healthy rats. Ferreira et al. [58] compared the effect of dry leaves of *Stevia rebaudiana* in both diabetic and healthy rats, reaching the conclusion that the stevia glycosides had no effect on the blood glucose reduction in normal conditions. Chen et al. [17] also reached similar conclusions as Suanarunsawat et al. [18] and Ferreira et al. [58], indicating that probably the physiological mechanism might be that stevioside reduces the phosphoenol piruvate carboxilase activity. Gregersen et al. [59] concluded that *Stevia rebaudina* reduced the levels of postprandial glucose in subjects with diabetes type II. All these studies seem to indicate that the possible hypoglycemic effect of *Stevia rebaudiana* is observed when diabetes occurs.

Concerning seric levels of triglycerides in male Wistar rats (Fig 6C) it was observed that the control group ingesting glucose sweetened drink had statistically higher values (189–245 mg/dL) than the blank group (62–117 mg/dL). To this respect, Samuel et al. [9] indicated that Jeppesen et al. [60] were the first researchers to show that both stevioside and steviol (1 nmol/L to 1 mmol/L) dose-dependently enhance insulin secretion from incubated mouse islets in the presence of glucose (*P<*0.05). The insulinotropic effects of stevioside and steviol were critically dependent on the glucose concentration and occurred at ≥8.3 mmol glucose/L (*P* < 0.05). To determine if stevioside and steviol act directly on pancreatic β cells, the β cell line INS-1 was used. Both stevioside and steviol potentiated insulin secretion from INS-1 cells (*P* < 0.05).

Park et al. [61] found that male mice from strain C57BL/6J fed with a fat-rich diet supplemented with *Stevia rebaudiana* extracts presented lower seric levels of triglycerides than those mice that consumed the fat-rich diet without it. Ritu and Nandini [62] observed a reduction of in seric levels of triglycerides in human patients with diabetes type II.

For the female Wistar rats, again, the control group drinking sweetened water with glucose had the highest values of triglycerides in blood serum (167–235 mg/dL). The other two groups had no statistical difference: Blank group (85–154 mg/dL) and the group drinking infusion of *Stevia rebaudiana* dried leaves (78–151 mg/dL). Sharma et al. [63] concluded that extracts of *Stevia rebaudiana* could reduce triglycerides in women that had hypertriglyceridemia. Thus, maybe new experiments should consider the evolution of the model animals to the adulthood and old age, especially the last one, when most of these types of metabolic diseases appear.

Cholesterol concentrations were not affected by the inclusion of the dry leaves of *Stevia rebaudiana* infusion or by the glucose added to the control drink independently of the sex (Fig 6E and 6F). These results agree with those of Ritu and Nandini [62], who observed that *Stevia rebaudiana* did not alter the levels of total cholesterol of patients with diabetes type II.

Going to the analysis of the hormones studied, the three male Wistar rats groups showed no differences in insulin concentrations in blood serum. Suanarunsawat et al. [18] found that the administration of crude extract of *Stevia rebaudiana* and of its stevioside did not cause any changes in insulin concentration levels for male Wistar rats. However, the same authors reported that when they were inducing diabetes through streptozotocine there was an increase in the seric levels of insulin after the 8 weeks of both crude extract of *Stevia rebaudiana* and stevioside dosification.

In female Wistar rats there were statistical differences in the insulin seric levels between the glucose control group and the blank one, being the highest ones. The group that drank the *Stevia rebaudiana* infusion was in the middle insulin values being neither different from the glucose control group nor from the blank group. Jeppesen et al. [60] concluded that the stevioside was able to stimulate *in vitro* insulin in β INS-1 cells. Continuing in this research, Jeppesen et al. [64] observed a reduced insulin response in the placebo group (P<0.05), whereas the insulin concentration was maintained in the stevioside group, suggesting that steviol glycosides may have a positive effect on β cell function in subjects with type 2 diabetes.

Glucagon has opposite effects to insulin, since it is produced when the levels of glucose diminish [65–67]. During fasting the levels of glucose decrease promoting the secretion of glucagon. When rats have similar glucose concentrations at fasting it was to be expected that levels of glucagon in blood serum were similar too, as it happened in this experiments (Fig 7C and 7D). Suanarunsawat et al. [18] reported that, after 8 weeks of administering crude extract of *Stevia rebaudiana* the rats had no changes in the levels of glucagon. However, with the administered stevioside a slight but significant increase in the levels of glucagon was observed. According to these results, this research (Fig 7C and 7D) and Suanarunsawat et al. [18], it may be sugested that the effect on glucagon levels depends upon the administration of pure stevioside and not with the crude extract of dried leaves of *Stevia rebaudiana*.

For leptin levels in blood serum, the three male Wistar rats groups (Fig 7E) showed higher values with respect to the female rats but were not statistically different among them. Mulet et al. [68] and Priego et al. [69] indicated that, contrary to what was observed in humans, female rats have the lowest leptin levels. In this research they had average values around 1.75–2.58 ng/mL for female rats *versus* 4.96–5.81 ng/mL for male rats. Figlewicz et al. [70] carried out experiments with male rats during 10 weeks and administered stevia, agave, fructose, and high fructose maize syrups water solutions to them. They found that there were no leptin levels changes for stevia water solutions.

In the female Wistar rats in the control group drinking water sweetened with glucose, the leptin levels were statistically higher with respect to the other two groups, stevia and blank (Fig 7F). As the levels of circulating leptin directly reflect the amount of stored fat tissue [71], and glucose being the metabolic precursor for lipogenesis, this slightly but significant increase of the leptin levels indicated a higher accumulation of fatty tissue. Statistically, the blank and stevia groups showed similar values of circulating leptin.

Concerning ghrelin, neither the male Wistar rat groups (Fig 7G) nor the female ones (Fig 7H) had significant differences in ghrelin concentrations. Ghrelin turns back to its basal levels from the fourth hour after food ingestion [72]. Considering that blood samples were taken after a 12-hour fasting, it would be normal to find no significant differences in the ghrelin levels. This hormone is closely related to appetite and the changes in the ghrelin levels are cyclic.

The GIP hormone (glucose dependent insulinotropic peptide) is related to the insulin liberation, as well as to the lipids metabolism enhancing lipogenesis [73]. The female specimens in average presented slightly higher levels of GIP, 31.6–36.5 *versus* 30.1–35.1 pg/mL in their male counterparts. For male rats there were no intergroup significant differences (Fig 7I). For the female specimens, glucose and *Stevia rebaudiana* groups presented lower levels of GIP than the blank group (Fig 7J), due to the fact that the hormone GIP depends on glucose concentration [73] With respect to the *Stevia rebaudiana* groups, results agree upon the reported data of Fujita et al. [74]. These researchers concluded that glucose but not artificial sweeteners (saccharin, acesulfame potassium, d-tryptophan, sucralose, or stevia) *in vivo* stimulate GIP. For these experiments in both for male and female rats, changes observed in the levels of GIP *(*Fig 7I and 7J) did not correspond to the changes in insulin levels (Fig 7A and 7B). The effects of sweeteners on the gut microbiota have been studied in recent years [75,76].

However, their relationship with the hormones GIP and GLP-1 (glucagon-like peptide-1) are still under study [73,77]. These findings may become a future subject for new experiments.

## Conclusions

The consumption of dry leaves infusions of *Stevia rebaudiana* for 120 days had no significant impact on body mass gain of male and female Wistar rats after weaning, showing similar responses to the blank group drinking plain water and the control group drinking glucose sweetened water. These results do not support the idea that *Stevia rebaudiana* consumption might promote body mass reductions. In young and healthy rats its effect was the same as that produced by plain drinking water or even water sweetened with glucose. Other therapeutic effects such as the reduction of blood serum glucose results were similar to those of Chen et al. [17], Ferreira et al. [58], and Suanarunsawat et al. [18]. It may be concluded that for young and healthy rats, the consumption of crude extracts of *Stevia rebaudiana* did not show negative effects on the normal blood serum levels of glucose, triglycerides, cholesterol, and the hormones insulin, glucagon, leptin, ghrelin, and glucose dependent insulinotropic peptide, GIP. It is probable that its possible positive effects are detected in pathological conditions such as diabetes type II [9], triglyceridemia [63], and other metabolic problems. For this reason, it is considered very important to continue with the evaluation of the safety and goodness of *Stevia rebaudiana* extracts.

### Official approval for animal experiments

Procedures were done using the guidelines of the National Research Council for the Care and Use of Laboratory Animals of the National Research Council Guide [36] as well as the existing Mexican legislation [37]. This study’s protocol was approved by the Institutional Ethics Committee for the Care and Use of Laboratory Animals (*CICUAL* in Spanish), of the Faculty of Chemistry of the National Autonomous University of Mexico (*Facultad de Quimica*, *UNAM*, in Spanish).

### Research highlights

The aim of this research is to corroborate the effect of dry leaves of *S*. *rebaudiana* in the drinking water of an animal model, Wistar rats of both sexes, male and females, in the first stage of growth after weaning, equivalent to infancy and youth. No negative effects on body mass gain were found when ingesting this stevia infusion compared with two control groups consuming drinking water with glucose and plain water during 120 days. Some interesting results in some blood serum biochemical parameters were also found.

## Supporting information

S1 Raw data (ZIP)

## Abbreviations

ANOVA: *analysis of variance*
CI: *confidence interval*
CONAHCYT: *Consejo Nacional de Ciencia y Tecnología*
CICUAL: Institutional Committee for the Care and Use of Laboratory Animals (*Comité Institucional para el Cuidado y Uso de Animales de Laboratorio* in Spanish)
n: *sample size*
h: *hour*
LD: *low density lipoproteins*
UNAM: *Universidad Nacional Autónoma de México* in Spanish
